# Multidrug-resistant ventilator-associated pneumonia (VAP) before and during the COVID-19 pandemic among hospitalized patients in a tertiary-care private hospital

**DOI:** 10.1017/ash.2023.293

**Published:** 2023-09-29

**Authors:** Alec Ann Alissa Aligui, Cybele Lara Abad

## Abstract

**Background:** Data on the incidence and outcome of ventilator-associated pneumonia (VAP) and multidrug-resistant VAP (MDR-VAP) among COVID-19 patients are limited. We compared the incidence and incidence density (ie, number of VAP per 1,000 ventilatory days) of MDR-VAP prior to and during the COVID-19 period in an urban, tertiary-care hospital. **Methods:** A retrospective study was conducted to compare the incidence, profile, and outcomes of patients with MDR-VAP during the pre–COVID-19 period (2018–2019) and during the COVID-19 pandemic (2020–2021). **Results:** In total, 80 (22%) of 362 patients developed VAP and were included in the cohort: 27 (33.75%) from the pre–COVID-19 period and 53 (66.25%) from the COVID-19 period, respectively. Most were male [20 (74%) of 27 vs 34 (64%) of 53], with median ages of 66 years (range, 35–90) and 67 years (range, 32–92) in the pre–COVID-19 and COVID-19 periods, respectively. Comorbidities were similar between the 2 periods, except for cardiovascular disease (14 vs 11; *P* = .005) and chronic lung disease (14 vs 9; *P* = .0012), which decreased significantly from the pre–COVID-19 period to the COVID-19 period. Only 15 (56%) of 27 versus 37 (70%) of 53 patients developed MDR-VAP during the pre–COVID-19 and COVID-19 period, with incidence densities of 19.3 of 1,000 and 27.8 of 1,000 ventilator days (*P* = .0371), respectively. The median length of stay prior to VAP for the pre–COVID-19 and COVID-19 periods were 17 and 10 days, respectively (*P* < .0001). Extended-spectrum β-lactamase (ESBL) resistance increased significantly from 1 (3.7%) of 27 before COVID-19 to 15 (28.3%) of 53 during the COVID-19 period. Carbapenem-resistant Enterobacteriaceae (CRE) resistance was higher before COVID-19 than during the COVID-19 period: 15 (56%) of 27 versus 10 (19%) of 53. In both periods, *Klebsiella pneumoniae* and *Acinetobacter baumannii* were the most common pathogens isolated. Mortality was high in both periods at 93% and 83%, respectively. Only female sex was associated with MDR-VAP in the COVID-19 period on multivariate analysis (OR, 3.47; 95% CI, 1.019–11.824; *P* < .047). **Conclusions:** The frequency of VAP and MDR-VAP increased during the COVID-19 period, despite a shorter median hospital stay. Mechanisms of resistance differed in the pre–COVID-19 and COVID-19 periods. Mortality with VAP was extremely high. The factors associated with increased risk of VAP and COVID-19 need to be studied further, and measures to prevent VAP should be prioritized.

**Figure:**
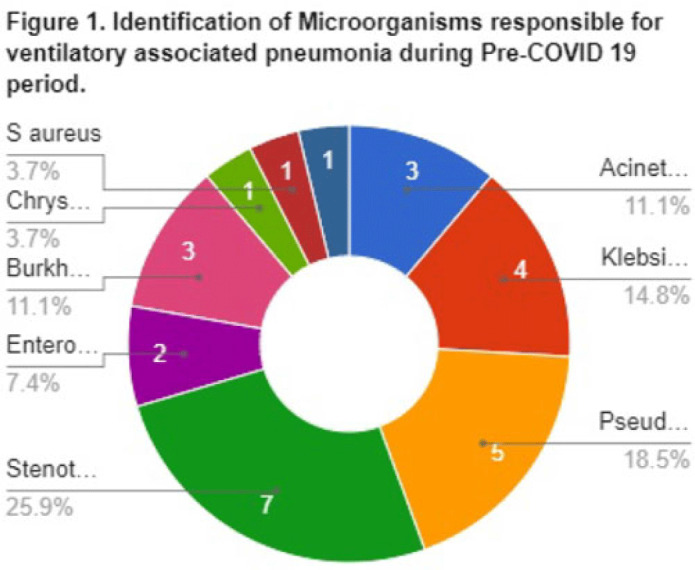

**Figure:**
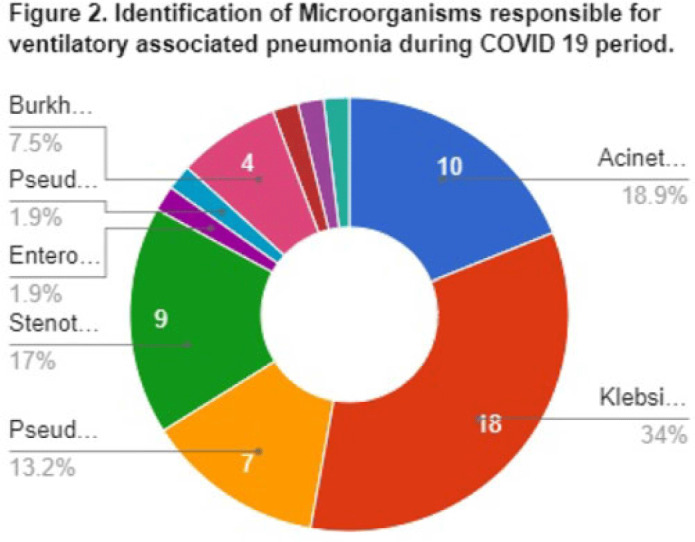

**Disclosures:** None

